# Conservative tibiotalocalcaneal fusion for partial talar avascular necrosis in conjunction with ankle and subtalar joint osteoarthritis in Kashin–Beck disease: A case report

**DOI:** 10.1097/MD.0000000000016367

**Published:** 2019-07-19

**Authors:** Liubing Li, Ying Wang, Zhenhua Zhu, Jupu Zhou, Shuyuan Li, Jianzhong Qin

**Affiliations:** a Department of Orthopaedics; b Department of General Surgery; c Department of Orthopaedics; d Department of Orthopaedics, the Second Affiliated Hospital of Soochow University, Suzhou, China; e The Foot and Ankle Association, Inc. Baltimore, MD; f Department of Orthopaedics, the Second Affiliated Hospital of Soochow University, Suzhou, China.

**Keywords:** avascular necrosis, Kashin–Beck disease, osteoarthritis, tibiotalocalcaneal fusion

## Abstract

**Rationale:**

Kashin–Beck disease (KBD) is known for some typical characters like finger joint enlargement, shortened fingers, and dwarfism. However, Avascular necrosis (AVN) of the talus in KBD has rarely been reported in the literature. Here, we reported on a KBD patient presented with partial AVN of the talus in conjunction with ankle and subtalar arthritis.

**Patient concerns:**

A 50-year-old woman presented with severe pain and limited range of motion in her left ankle and subtalar joint while walking for 2 years. She had been walking with the aid of crutches for many years. Conservative treatment with rigid orthosis and activity restriction could not help reduce the pain in the left foot.

**Diagnoses:**

Radiographs demonstrated that partial AVN was developed in the body of the talus and arthritis was viewed in the left ankle and subtalar joint. Hence, we established the diagnosis of partial talar AVN in conjunction with ankle and subtalar arthritis.

**Interventions:**

A conservative tibiotalocalcaneal fusion attempting to preserve as much viable talar body as possible was performed using a humeral locking plate and 2 cannulated compression screws.

**Outcomes:**

Bone union proved by CT scan and a good alignment of the left limb were achieved at 4-month follow-up postoperatively.

**Lessons:**

Partial AVN of the talus along with ankle and subtalar arthritis in KBD patients has rarely been reported as it is not a common characteristic of KBD in clinical practice. Conservative tibiotalocalcaneal fusion could help preserving much more viable talar body, maintaining most structural integrity of the ankle joint, and achieving a stable and plantigrade foot postoperatively.

## 1 Introduction

Kashin–Beck disease (KBD) is an endemic, symmetrical disease characterized by degeneration and necrosis of the epiphyseal growth plate and the articular cartilage.^[1]^ Although 3 major environmental factors including endemic selenium deficiency, cereal contamination by mycotoxin-producing fungi and high humic acid levels in drinking water have been supposed to contribute to KBD, the etiology has not been clearly established. In China, KBD, also known as “Big Bone Disease”, is mainly distributed in broad areas extending from the northeast to southwest, involving over 377 counties of 13 provinces or autonomous regions.^[2]^ Up to 2013, it was estimated that about 0.64 million patients were affected by KBD and 1.16 million at risk. Children at the age of 7 to 13 years are most commonly suffered from KBD characterized by chondrocyte necrosis and apoptosis of growth plate cartilage and articular cartilage.^[2]^

Clinical syndromes of KBD mainly include finger joint enlargement, shortened fingers, and dwarfism. According to the new diagnostic criteria (WS/T207–2010), KBD was classified as 3 stages.^[3]^ Stage I KBD is defined as enlarged finger joints, limited range of motion (ROM) and pain in the joints of the limbs, stage II as shortened fingers and clinical symptoms of first stage, and stage III as dwarfism and clinical symptoms of stage II.^[3]^

Previous reports on KBD in the literature mainly focused on enlarged finger joint deformities, shortened fingers, and dwarfism, and very few articles have reported changes in foot and ankle. In fact, the most frequently affected joints are ankles, knees, wrists, and elbows leading to atrophied muscles and even disability in daily life activities.^[4]^ It is reported that ankle joint damage is involved in 69.3% of the patients with KBD while knee in 12.5% and hip joint in 2%.^[4]^ Since the talus bears the most of the body weight, it is reasonable to speculate that the chondrocyte necrosis and apoptosis of articular cartilage of the talus would become more common than those in other joints, which will progressively lead to talus depression, avascular necrosis (AVN), and arthritis in adjacent joints. To the best of our knowledge in the literature, rare articles have reported on partial talar AVN and radiographic changes in patients with KBD.^[5]^ In this study, we presented a rare case with KBD of partial AVN located at the superior portion of the talar body in conjunction with ankle and subtalar osteoarthritis, and a successful treatment through conservative tibiotalocalcaneal fusion with a humeral locking plate and cannulated compression screws.

## 2 Case report

A 50-year-old woman presented to our clinic with a 2-year history of severe left ankle pain, in particular when walking on uneven ground. The pain started in her early 20 s with moderate pain in her right knee. She had been walking with the aid of crutches for many years. Without the crutches, she could walk no more than 100 m. She was conservatively treated for more than 1 year with rigid orthosis and activity restriction. Even after this conservative treatment, she still suffered persistently from the left foot. She had mild tenderness in the right ankle as well. For this visit, she merely wanted to address left ankle issues. There was no history of systemic steroid or chemo-therapeutic agent use, trauma, or alcohol addiction.

**Figure 1:**
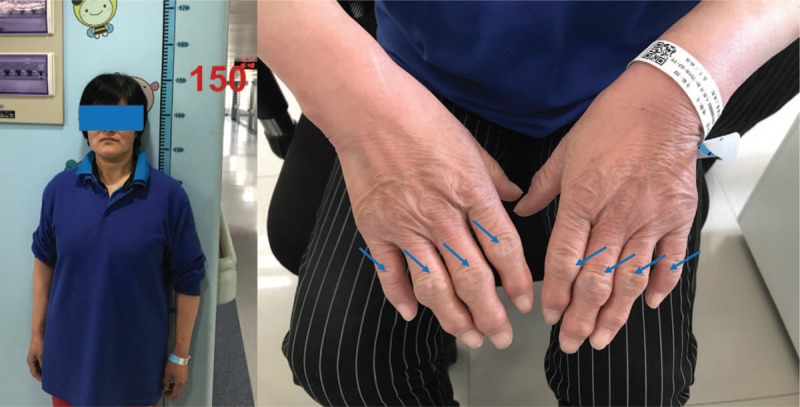
(A) She was dwarf, just over 1.5 meters tall. (B) She had enlarged distal and proximal interphalangeal joints and shortened fingers in both of her hands.

On physical examination, she was just over 1.5 m tall. There were enlarged distal interphalangeal (DIP)/proximal interphalangeal (PIP) joints and shortened fingers in both hands (Fig. 1). There were severe tenderness and moderate swelling in the left ankle with 0° of dorsiflexion and 5° of plantar flexion, and 5° of inversion and 5° of eversion in the left subtalar joint. The American Orthopaedic Foot and Ankle Society (AOFAS) ankle/hindfoot score was 34 and visual Analysis Score (VAS) was 8. Serum rheumatoid and inflammatory tests were negative.

**Figure 2:**
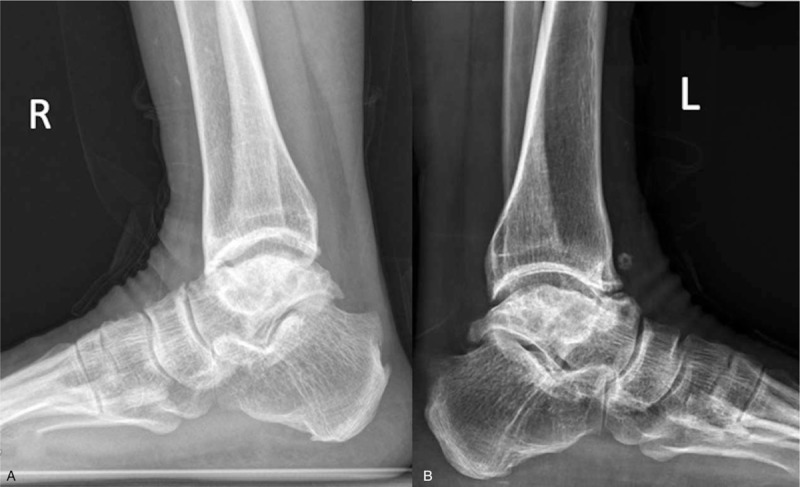
The radiographs demonstrated partial talar avascular necrosis (AVN) with collapse and sclerotic changes of the talar body on both sides. Characteristic calcaneal shortening deformity was demonstrated on both ankle radiographs (A–B). Osteophytes and loose bodies were demonstrated in the left ankle joint (B).

**Figure 3:**
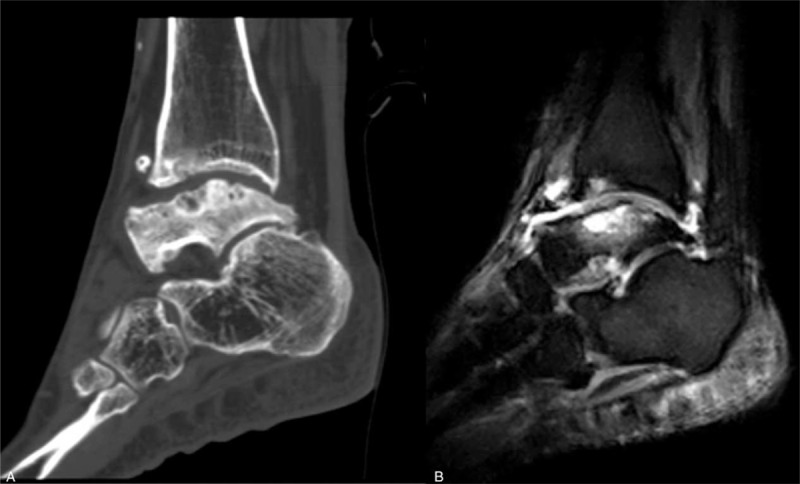
(A) The computerized tomography (CT) scan demonstrated that cystic lesions were developed at the dome of the talus and distal tibia. (B) Magnetic resonance imaging (MRI) showed extensive areas of high-signal intensity in the body of the talus and nonspecific inflammation in the left ankle and subtalar joint on T2-weighted images.

Radiographs showed partial talar AVN with collapse and sclerotic changes of the talar body on both ankle joints. Osteophytes and loose bodies were demonstrated in the left ankle joint. Significant degenerative arthritis and subchondral sclerosis were found in the left ankle and subtalar joints. Similar subchondral sclerosis and partial talar depression were demonstrated in the right ankle radiographs (Fig. 2). Computerized tomography (CT) scan revealed that cystic lesions were developed at the dome of the talus and distal tibia in the left ankle joint. Magnetic resonance imaging (MRI) showed extensive areas of high-signal intensity in the body of the talus and nonspecific inflammation in left ankle and subtalar joint on T2-weighted images (Fig. 3). Regrettably, she did not agree to do a CT or MRI scan in the right ankle. Therefore, the difference between both sides cannot be obtained in detail.

**Figure 4:**
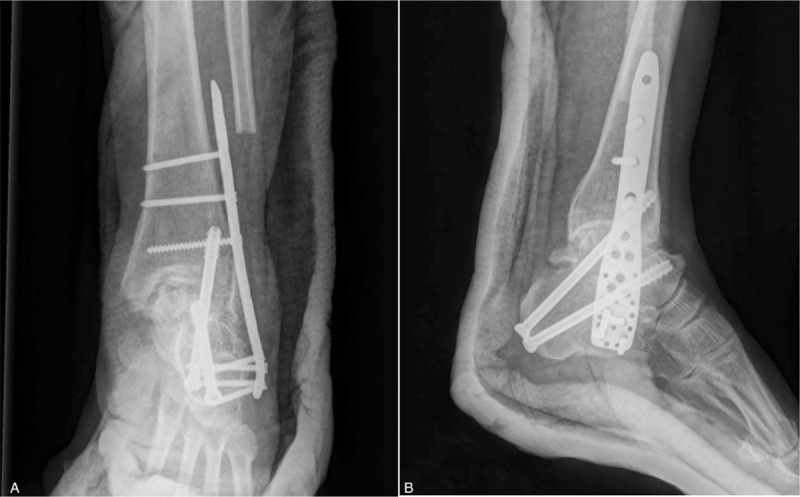
(A) The proximal humeral internal locking plated was inverted and fixed with a minimal invasive plate osteosynthesis (MIPO) technique. (B) Two 7.0-mm cannulated screws were inserted from the tuberosity of the calcaneus, aiming at the talar head and anterior malleolus of the distal tibia.

According to the new diagnostic criteria (WS/T207–2010) in KBD,^[3]^ this patient was diagnosed as KBD with partial AVN of the talar body in conjunction with end stage osteoarthritis in left ankle and subtalar joints, and the same diagnosis in right ankle joint. Since she mainly complained of severe tenderness in left ankle and subtalar joint, this patient merely wanted to address left ankle issues during this visit. A conservative tibiotalocalcaneal fusion was performed through anteromedial and lateral approaches at the ankle joint in supine position. A lateral incision was made first along the distal fibular, starting about 7.0 cm proximal to the tip of lateral malleolus then curving anteriorly in the direction of sinus tarsi. An osteotomy was made 7.0 cm proximal to the tip of lateral malleolus after subperiosteal detachment keeping peroneal tendon sheath intact. The distal fibular was resected with an oscillating saw and used as bone graft. The talocrural and subtalar joints were fully exposed. Osteophytes and loose bodies were removed and partial necrotic talar body was conservatively resected in order to preserve as much viable talar body as possible and maintain most structural integrity of ankle joint. Secondly, a 5.0-cm slightly curved incision was made on the anteromedial aspect of the ankle, just anterior to the tip of the medial malleolus. Through 2 incisions, the articular cartilages of talocrural and subtalar joint were curetted and prepared for fusion. All subchondral bone surfaces were perforated with a 2.0-mm Kirschner wire to stimulate bone marrow outflow. A proximal humeral internal locking plate (Kanghui, Changzhou, China) was placed upside down laterally. The proximal portion of the plate was fixed to the calcaneus with 4 locking screws in multiple planes, while its distal portion was inserted into the proximal portion of the distal tibia with a minimal invasive plate osteosynthesis (MIPO) technique. Two 7.0-mm cannulated screws were inserted from the tuberosity of the calcaneus, aiming at the talar head and anterior malleolus of the distal tibia to strengthen the fixation (Fig. 4). The patient was maintained non-weight bearing for 6 weeks postoperatively and was allowed to progressively bear weight in an AirCast walking brace until bone union was confirmed 4 months postoperatively.

**Figure 5:**
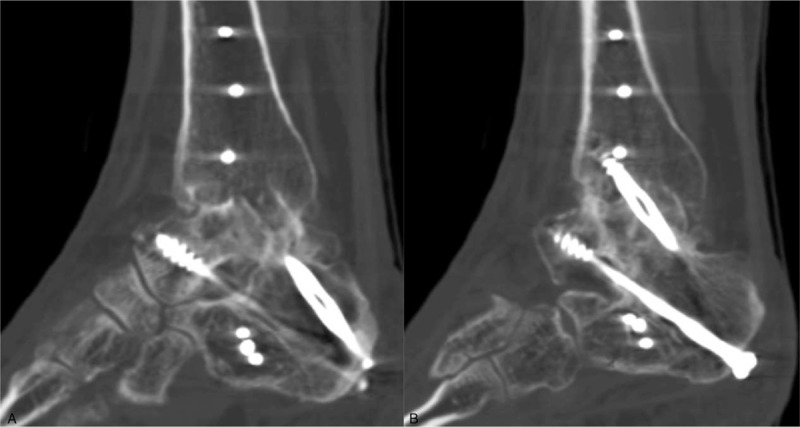
The CT scan taken at 4-month follow-up after surgery demonstrated bone union and a good alignment of the foot.

A CT scan taken at 4 months after surgery showed solid bone union and good alignment of the foot (Fig. 5). No significant length discrepancy was observed between 2 legs. The AOFAS ankle/hindfoot score and VAS was 73 and 3 at 7-month follow-up after surgery, respectively.

## 3 Discussion

According to the 2010 diagnostic criteria^[3]^, this patient was typically classified as stage III in KBD, encompassing enlarged finger joint, shortened fingers, dwarfism and severe tenderness, and limited ROM of her ankle joints. She was diagnosed as KBD with partial AVN of the talar body in conjunction with end stage osteoarthritis in the left ankle and subtalar joints, and the same in right side. Since she had been complaining of severe pain on the left side, we merely addressed left ankle and subtalar issues during this visit.

AVN of the talus frequently occurs after talar neck fracture with dislocation (Hawkins classification II-IV). Generally, the AVN of the talus was thought of as an “all-or-nothing” process.^[6]^ However, some authors believed that this “all-or-nothing” concept was derived from the limitation technique of plain radiography to find out focal changes in the talus.^[7]^ Partial AVN of the talus was first reported by Peterson.^[8]^ In 2003, Tehranzadeh and colleagues reported 3 cases of partial Hawkins sign with all of the 3 cases having similar ischemic changes on the dome.^[9]^ Babu et al^[7]^ reported a series of cases who developed partial AVN of the talar body after displaced fracture of the talar neck. They suggested that the predominant positions of avascular segments were located at the anterior lateral and superior portion of the talar body, which was corresponding to regional damage to the blood supply of talar body. Partial AVN of the talus with collapse was found at the superior portion of the talar body in our KBD patient, which was significantly different from what literatures reported in talar neck fracture cases. This may be due to the different pathophysiological mechanisms. As we all know, KBD is of environmental origin.^[10]^ Among those series of environmental risk factors, the selenium deficiency and the cereal contamination and high humic acid levels in drinking water are supposed to be the major environmental risk factors.^[2,11]^ The epiphyseal growth plate and the articular cartilage are most commonly affected by the aforementioned risk factors in KBD patients. The chondronecrosis has been revealed to be the characteristic changes occurring in the deep zone of the articular cartilages and epiphyseal growth plate cartilages in KBD, which can result in a disturbed endochondral ossification and necrosis, and even cause early closure of the epiphyseal growth plate.^[12,13]^ The younger the onset of symptoms, the more severe the deformities of the limbs. In this patient, we suspected that the cartilage of the talus could not bear the most of the body weight progressively, which led to the collapse and AVN of the talus and adjacent joint arthritis.

Management of AVN of the talus is always a frustrating and embarrassing challenge to the surgeons, even to the very good surgical hands. Gross et al^[14]^ reviewed the literature and made the treatment recommendations for AVN of the talus. Surgical treatment options are mostly indicated to patients who have pain and deformity in conjunction with arthrosis of the ankle, subtalar joint, and osteonecrosis of the talus.^[15]^. According to the literature, various treatment strategies for talar AVN can be grouped into 2 categories, joint-sacrificing procedures, such as talectomy and arthrodesis, and joint-sparing procedures, such as conservative treatment, core decompression, and total ankle replacement.^[14,16]^ Although aforementioned strategies have been reported in the literature, there is currently no consensus with regard to the ideal options to talar AVN. Conservative treatment and core decompression have been suggested as alternatives for treatment of AVN of the talus prior to collapse.^[17]^ Protected weight bearing and electric shock wave therapy (ESWT) were recommended for early talar AVN. If failed, core decompression could be an attractive option. Arthrodesis was recommended as a salvage procedure.

In this patient, she had severe pain and limited ROM in left ankle and subtalar joints. On radiographs, significant degenerative ankle and subtalar arthritis with obvious marginal osteophytes and loose bodies were identified according to Kellgren–Lawrence classification scale for osteoarthritis severity.^[18]^ Partial AVN of the talar body and ankle and subtalar arthritis were demonstrated on CT and MRI scans as well. Therefore, our purpose is to preserve as much viable talar body as possible, maintain most structural integrity of left ankle joint and achieve a stable and plantigrade foot postoperatively.

Several strategies, such as external fixation device, intramedullary nail, and locking plate, have been reported on tibiotalocalcaneal fusion in the literature. External fixation devices are optimal for severe osteoarthritis and rigid ROM in the foot and ankle, and allowing limb lengthening as well.^[19,20]^ However, they are not indicated for patients with osteoporosis or poor osteogenesis and also correlated with a high risk of infection.^[21]^ Although tibiotalocalcaneal fusion with retrograde intramedullary nailing has been proven to have a higher union rate, many complications have been reported in relation to this procedure, including neurovascular injury, delayed unions, non-unions, even limb amputation.^[22,23]^ Locking plates have been generally used in tibiotalocalcaneal fusion, such as humeral and femoral locking plates and high successful union rates have been reported as well in the literature. However, no paper has been published on the use of a locking plate in partial AVN of talar body along with ankle and subtalar osteoarthritis in KBD patients. The proximal humeral locking plate could not only provide rigid fixation by placing several screws into the calcaneus at various angles, but more easily obtain a desirable plantigrade position: neutral dorsiflexion, neutral to 5° valgus, and external rotation similar to that of the contralateral limb. Additionally, 2 percutaneous cannulated compression screws were inserted from the tuberosity of the calcaneus, aiming at anterior portion of the distal tibia and talar head, respectively. In this case, we attempted to preserve as much viable talar body as possible conservatively. It was proved that bone union and good alignment were achieved at the 4-month follow-up.

In conclusion, early closure of the epiphyseal growth plate and chondrocyte necrosis are the main pathologic manifestation in KBD, which can cause multiple, symmetrical joint damages, including finger, ankle, knee, and hip joints. The axial load of body weight on the ankle joint could probably accelerate the damage of articular cartilage of the talus which leading to potential talus depression, AVN and its adjacent joint arthritis. For patients with KBD of partial AVN of the talar body along with ankle and subtalar arthritis, conservative tibiotalocalcaneal fusion with a humeral locking plate and cannulated compression screws could help preserving much more viable talar body, maintaining most structural integrity of the ankle joint, and achieving a stable and plantigrade foot.

## Acknowledgments

The authors thank this patient for the consent to publish her story and pictures in the journal.

## Author contributions

**Investigation:** Liubing Li, Ying Wang, Jupu Zhou.

**Methodology:** Jianzhong Qin.

**Resources:** Zhenhua Zhu.

**Writing – original draft:** Liubing Li, Ying Wang.

**Writing – review & editing:** Shuyuan Li, Jianzhong Qin.
