# Conservative tibiotalocalcaneal fusion for partial talar avascular necrosis in conjunction with ankle and subtalar joint osteoarthritis in Kashin–Beck disease: A case report: Erratum

**DOI:** 10.1097/MD.0000000000016979

**Published:** 2019-08-23

In the article, “Conservative tibiotalocalcaneal fusion for partial talar avascular necrosis in conjunction with ankle and subtalar joint osteoarthritis in Kashin–Beck disease: A case report”,^[1]^ which appears in Volume 98, Issue 29 of *Medicine*, the following funding information should be noted:

This study was funded by Science and Technology Projects of Suzhou Sanitary Bureau (SYS2018052) and Suzhou Municipal Sports Bureau Sports Research Bureau (TY2016-203).
